# Adherence to the 24 h Movement Guidelines and Associated Factors Among First-Year University Students in Spain: UNILIFE-M Study at Universidad Loyola Andalucía

**DOI:** 10.3390/healthcare14203415

**Published:** 2026-10-13

**Authors:** José Francisco López-Gil, Samuel Manzano-Carrasco, José Adrián Montenegro-Espinosa, María Mendoza-Muñoz, Fiorella Quiroz-Cárdenas, Alejandro Lara-Bocanegra, Aline Josiane Waclawovsky, Debora Tornquist, Andrea Camaz Deslandes, Rodrigo Yáñez-Sepúlveda, Felipe Barreto Schuch, Lucille Ridgell, Camila Miño, Dong Keon Yon, Pablo Galan-Lopez

**Affiliations:** 1 School of Medicine, Universidad Espíritu Santo, Samborondón 092301, Ecuador; 2 Faculty of Health Sciences, Universidad Tecnológica Atlántico Mediterráneo—UTAMED, 29590 Málaga, Spain; 3 Department of Communication and Education, Universidad Loyola Andalucía, 41704 Dos Hermanas, Spain; smanzano@uloyola.es (S.M.-C.); mariamendozam10@gmail.com (M.M.-M.); pgalan@uloyola.es (P.G.-L.); 4 Universidad Autónoma del Paraguay, Asunción 001010, Paraguay; josemontenegro733@gmail.com; 5 Vicerrectoría de Investigación y Postgrado, Universidad de Los Lagos, Osorno 5290000, Chile; fiorella.quiroz@yahoo.com; 6 Department of Health Research, Icen Cognis, Carretera General, 32, 38370 La Matanza de Acentejo, Spain; 7 Departamento de Educación Física y Deporte, Universidad de Sevilla, 41013 Seville, Spain; alejandrolarboc@us.es; 8 Psychiatry Institute, Federal University of Rio de Janeiro, Rio de Janeiro 21941-901, Brazil; alinejosianew@gmail.com (A.J.W.); adeslandes@ipub.ufrj.br (A.C.D.); felipe.schuch@ufsm.br (F.B.S.); 9 Department of Sports Methods and Techniques, Federal University of Santa Maria, Santa Maria 97105-900, Brazil; debora.tornquist@gmail.com; 10 Faculty of Education and Social Sciences, Universidad Andrés Bello, Viña del Mar 8370035, Chile; rodrigo.yanez.s@unab.cl; 11 Department of Sport Sciences, Faculty of Sport and Health Sciences, Fit Generation Research Institute, AD500 Andorra la Vella, Andorra; 12 Faculty of Health Sciences, Universidad Autónoma de Chile, Providencia 7500912, Chile; 13 Food and Mood Centre, Institute for Mental and Physical Health and Clinical Translation (IMPACT), Deakin University, Geelong 3220, Australia; 14 Institute for Physical Activity and Nutrition (IPAN), Deakin University, Geelong 3220, Australia; 15 Carver University, Orlando, FL 32804, USA; lucilleridgell@gmail.com; 16 Universidad Internacional para el Desarrollo (UNINDE), 06005 Badajoz, Spain; 17 Center for Digital Health, Medical Science Research Institute, Kyung Hee University Medical Center, College of Medicine, Kyung Hee University, Seoul 02447, Republic of Korea; yonkkang@gmail.com; 18 Department of Pediatrics, Kyung Hee University Medical Center, Kyung Hee University, Seoul 02447, Republic of Korea

**Keywords:** young adults, behavior patterns, university education, public health promotion, lifestyle risk factors

## Abstract

**Highlights:**

**What are the main findings?**

- Only 21.2% of first-year students at this Spanish university (137/646 with complete guideline data) met all three 24 h movement guidelines for physical activity, sedentary behavior (total sitting and recreational screen time), and sleep, and 8.4% met none.
- In multivariable analyses, adherence was lower among females and students with a lifetime mental health diagnosis; associations for age >18 years and non-health sciences enrollment were borderline and not robust in sensitivity analyses.

**What are the implications of the main findings?**

- Integrated movement behaviors may warrant attention in university health promotion efforts, particularly among subgroups with lower observed adherence in this sample.
- The cross-sectional association with diagnosed mental health problems is consistent with co-occurrence of psychiatric symptoms and lower adherence, although the temporal direction cannot be established in this study.

**Abstract:**

**Background/Objectives:** University students are at a critical stage for establishing healthy lifestyle habits, yet little is known about their adherence to integrated 24 h movement guidelines that include physical activity, sedentary behavior, and sleep. We examined the proportion of students meeting the Canadian 24 h movement guidelines and associated factors among first-year students at a single Spanish university (UNILIFE-M). **Methods:** This cross-sectional analysis included 671 first-year students from Universidad Loyola Andalucía (Spain) participating in the UNIversity Students’ LIFEstyle Behaviours and Mental Health (UNILIFE-M) study. Combined guideline adherence used complete cases (n = 646). Behaviors were assessed with the Spanish Physical Activity Scale 2.1 (PAS-2.1S). Adherence required ≥150 min/week of moderate-to-vigorous physical activity, ≤8 h/day of total sedentary time including ≤3 h/day of recreational screen time (both sedentary criteria required), and 7–9 h/night of sleep. Multivariable logistic regression estimated adjusted odds ratios (complete cases on covariates, n = 644). **Results:** Overall, 137/646 students (21.2%) met all three guidelines, while 54 (8.4%) met none. Male sex was associated with higher odds of adherence (adjusted odds ratio [aOR] = 1.65; 95% confidence interval [95% CI] 1.10 to 2.47), and a lifetime mental health diagnosis was associated with lower odds (aOR = 0.28; 95% CI 0.08 to 0.72). Age > 18 years (aOR = 0.64; 95% CI 0.39 to 1.03) and non-health sciences enrollment (aOR = 0.69; 95% CI 0.46 to 1.03) showed borderline associations that were not robust across sensitivity analyses. **Conclusions:** In this single-university first-year sample, adherence to the 24 h movement guidelines was relatively low. Associations should be interpreted as cross-sectional correlates, not causal determinants.

## 1. Introduction

The university stage typically coincides with emerging adulthood (approximately 18–25 years), a developmental period marked by significant biological, psychological, and social transformations [1,2]. Neurodevelopmentally, the prefrontal cortex, responsible for impulse control, planning, and emotion regulation, continues to mature during this stage, increasing vulnerability to external stressors and influencing decision-making processes [1,3]. Simultaneously, emerging adults face increased autonomy, academic demands, identity exploration, and changes in social networks, all of which can disrupt established routines and challenge self-regulation [4].

Maintaining a healthy lifestyle is particularly important during this life stage, as it contributes to both immediate psychological well-being and long-term disease prevention [5]. Evidence suggests that health-related behaviors formed during emerging adulthood tend to track into later adulthood [6,7], underscoring the importance of promoting sustainable habits early on. However, the transition to university life is often associated with deteriorating health routines [8,9], highlighting the need for targeted efforts to foster healthier lifestyle patterns during this vulnerable period.

Traditionally, physical activity, sedentary behavior, and sleep have been studied as independent lifestyle components. However, examining these behaviors separately overlooks how they interact in daily time use. For this reason, research has adopted the concept of “24 h movement behaviors” [10]. This encompasses all movement-related activities, ranging from physical activity and sleep duration to sedentary behavior (including screen time), on a 24 h continuum [11]. According to these guidelines, a healthy routine for adults aged 18–64 years should include at least 150 min per week of moderate- to vigorous-intensity physical activity (MVPA), ensure 7–9 h of restful sleep, and limit time spent in sedentary behaviors to no more than 8 h per day, also restricting leisure screen time to no more than 3 h [12]. On this basis, studies have established an association between adherence to the 24 h movement guidelines and various health indicators across the lifespan [13,14,15,16].

A meta-analysis encompassing 387,437 participants aged 3 to 18 years across 23 countries revealed that a mere 2.68% adhered to the 24 h movement guidelines [17]. Compliance with these guidelines was notably lower among adolescents, particularly females. In Europe, only 9.62% of the sample adhered to all three recommendations, while 13.48% did not adhere to any, underscoring the low compliance with these guidelines. However, there is a paucity of literature concerning adults, particularly university students. A multi-center cross-sectional survey conducted by Pengpid and Peltzer [18] involving 3223 university students from Indonesia, Malaysia, Myanmar, Thailand, and Vietnam reported that 11.7% met all three 24 h movement guidelines. Another study by Zhang et al. [19] conducted among 1793 Chinese university students, found that 27.8% adhered to the 24 h movement guidelines, but there is a scarcity of studies in European students.

Regarding correlates of adherence to the 24 h movement guidelines, the systematic review by Rollo et al. [13] reported that, among adults, adherence was more likely among individuals who were younger, male, had a lower body mass index (BMI), higher educational attainment, were employed, had higher income, and did not have children. In more specific samples, Pengpid and Peltzer [18] found greater adherence among males (15.6%), individuals aged 22–30 years (17.0%), and those from lower-income countries such as Indonesia, Myanmar, and Vietnam (14.6%). Similarly, Bu et al. [20] observed that male university students, and those whose mothers held a master’s degree or above, were more likely to meet all three movement behavior recommendations, which was associated with lower anxiety symptoms compared with meeting fewer guidelines.

Although studies on adherence to 24 h movement guidelines have been conducted in countries such as Indonesia, Malaysia, Myanmar, Thailand, Vietnam, China, and Canada, none have specifically targeted Spanish first-year university students. This group, due to its transitional developmental stage and the physical, psychological, and social changes it undergoes, is particularly vulnerable to symptoms of depression and anxiety, which tend to increase throughout the university years. Moreover, cultural norms, academic pressures, and lifestyle patterns in Spain may influence movement behaviors and mental health outcomes differently compared to other countries, highlighting the need for context-specific research. Therefore, the aim of the present study was two-fold: first, to determine the proportion of adherence to the 24 h movement guidelines among first-year students at Universidad Loyola Andalucía (Spain); second, to identify associated factors (correlates) of that adherence.

## 2. Materials and Methods

### 2.1. Population and Study Design

The UNIversity Students’ LIFEstyle Behaviours and Mental Health (UNILIFE-M) [21] is a global prospective cohort study aimed at investigating the links between university student’s lifestyle choices and mental health symptoms throughout their academic careers. The study’s research framework was previously detailed in the UNILIFE-M study [21]. UNILIFE-M collects self-reported data through an online survey that assesses mental health symptoms such as depression, anxiety, mania, sleep disturbances, substance abuse, inattention-hyperactivity disorder, and obsessive-compulsive thoughts or behaviors, alongside lifestyle factors like diet, physical activity, substance use, stress management, social support, restorative sleep, the environment, and sedentary behavior over a period of 3.5 years. Participants from 84 universities in 27 countries were assessed upon entry in the 2023 and/or 2024 academic year and will be followed up at 1-, 2-, and 3.5-year intervals.

In this study, data was collected from Universidad Loyola Andalucía during the 2024 academic year, involving 923 participants, of whom 671 completed all the questionnaires. Participants who did not meet the inclusion criteria (130), those who left the questionnaire entirely blank (114), and those with one or more unanswered questions (8) were excluded. The study’s inclusion criteria required participants to be between 18 and 35 years old and to be enrolled as first-year students, specifically in their first semester, during the 2024 academic year. Students from various faculties and disciplines were eligible to participate. Those not meeting these criteria were excluded from the analysis. A team of trained psychologists administered the questionnaires to students during the initial weeks of classes using convenience sampling. Students from different faculties and specializations were recruited through online platforms (such as university websites, virtual classroom messages, and online reminders), mass emails, and visits to classrooms where first-semester courses were held to inform them about the study and seek their participation in completing the questionnaires, all in compliance with relevant data privacy laws and policies.

This study was reviewed and approved by the Research Ethics Committee of Universidad Loyola Andalucía (approval ID: 240605/CE24544; approved on 19 September 2024). All procedures involving human participants were conducted in accordance with the ethical standards of the institutional and/or national research committee and with the Declaration of Helsinki and its later amendments. Written informed consent was obtained from all participants prior to their inclusion in the study.

### 2.2. Variables

#### 2.2.1. Lifestyle

##### Twenty-Four-Hour Movement Behaviors

Twenty-four-hour movement behaviors were evaluated using the validated Spanish version of the Physical Activity Scale 2.1 (PAS-2.1S) [22], which measures physical activity across work/school, transportation, and leisure domains. The PAS-2.1S was not originally conceived within the UNILIFE-M protocol. However, in the present study, we included it as an additional instrument in the sample from Universidad Loyola Andalucía. This instrument consists of nine items: six focus on daily activities such as sleep duration, sedentary time, leisure activities, and commuting, while three assess weekly physical activity at light (LPA), moderate (MPA), and vigorous (VPA) intensity levels. Weekly moderate-to-vigorous physical activity (MVPA) was computed as the sum of MPA and VPA minutes without vigorous-intensity double-weighting. Meeting the MVPA guideline was defined as ≥150 min per week [12]. Consistent with the Canadian 24-Hour Movement Guidelines for adults aged 18–64 years [12], participants were classified as meeting the sedentary behavior recommendation if they reported ≤480 min/day (≤8 h/day) of total sedentary time and ≤180 min/day (≤3 h/day) of recreational screen time (both criteria required). Meeting the sleep recommendation was defined as 420–540 min/night (7–9 h/night). Participants were categorized based on the number of 24 h movement guidelines they met, ranging from 0 to 3, and were grouped into four categories: “none of the guidelines”, “one guideline”, “two guidelines”, and “all three guidelines”. Of 671 participants with baseline questionnaires, 25 had missing data on one or more guideline components, yielding an analytic sample of 646 for combined 24 h guideline proportion.

#### 2.2.2. Sociodemographic Information

Self-reported data were collected on age (in years), sex (male/female; biological sex as captured by the questionnaire item), sexual orientation (originally including heterosexual, homosexual, bisexual, pansexual, and other), marital status (original categories ranging from single to married, separated, divorced, or widowed), student accommodation (yes/no), employment status (yes/no), race/ethnicity (originally categorized into multiple groups), and university degree (based on academic program enrollment). For analytical purposes, the following dichotomous variables were created: age was grouped as “≤18 years old” or “>18 years old” (based on the sample median); sexual orientation was dichotomized as “LGBTQ+” (including homosexual, bisexual, pansexual, and other identities) or “heterosexual”; marital status was recoded into “single” or “non-single”; student accommodation and employment status were recoded as “yes” or “no”; and ethnic group was categorized as “Caucasian” or “non-Caucasian” (collapsed because of small cell sizes in non-Caucasian groups; n = 42; 6.3%). Degree programs classified as health sciences included Pharmacy, Nursing, Physiotherapy, Biotechnology, and Physical Activity and Sport Sciences (CAFYD), including related dual degrees; all other programs were classified as non-health sciences.

#### 2.2.3. Anthropometric Information

Anthropometric data were self-reported and included weight (kg) and height (m), from which BMI (kg/m^2^) was calculated. BMI categories followed World Health Organization (WHO) adult cut-offs: underweight, normal weight, overweight, and obesity [23]. For analysis, overweight/obesity was defined as BMI ≥ 25kg/m^2^ (15.8%, 106/669 with valid anthropometric data; median BMI 21.9 kg/m^2^).

#### 2.2.4. Mental and Physical Health Information

Participants were asked whether they had ever been diagnosed with a mental health problem or developmental disorder by a psychiatrist or psychologist. Responses were dichotomized into “yes” or “no” to create a binary lifetime clinician-diagnosis variable representing mental health diagnosis status (heterogeneous diagnoses without severity information). In addition, participants were asked to report whether they had ever been diagnosed with specific mental disorders (e.g., depression, anxiety, eating disorders) and physical conditions (e.g., asthma, diabetes, epilepsy, coeliac disease). These conditions were grouped into two broad categories: (a) mental health disorders: defined as having received a diagnosis of any of the following conditions: depression, anxiety, attention-deficit/hyperactivity disorder (ADHD), autism spectrum disorder (ASD), eating disorders, or other related mental health diagnoses; (b) physical health conditions: defined as having received a diagnosis of any chronic somatic disease, including cardiovascular, respiratory, metabolic, neurological, or gastrointestinal disorders.

### 2.3. Statistical Analyses

Descriptive statistics were computed for all study variables. The distribution of continuous variables (e.g., age, BMI, MVPA, sitting time, and sleep duration) was assessed using the Shapiro–Wilk test, density plots, and quantile–quantile (Q–Q) plots. All variables showed non-normal distributions (*p* < 0.05) and visual deviation from normality. Therefore, continuous variables are presented as medians with interquartile ranges (IQR), while categorical variables are summarized using frequencies and percentages.

To examine associations between sociodemographic and health characteristics and adherence to all three 24 h movement guidelines, we fitted a multivariable logistic regression model with simultaneous entry of a priori covariates (binomial GLM with logit link). Results are presented as adjusted odds ratios (aORs) with 95% confidence intervals (95% CIs). Primary models used complete cases on the outcome and covariates (n = 644; 136 events), reflecting two guideline-complete participants without valid BMI. Multicollinearity was assessed with variance inflation factors (all VIFs < 1.2). Because events were sparse among participants with a mental health diagnosis (4/64 met all three guidelines in the guideline-complete sample), we conducted a Firth penalized logistic regression sensitivity analysis. Age was modeled both as a median split (≤18 vs. >18 years) and as a continuous variable in sensitivity analyses. Additionally, Venn diagrams were generated to visually illustrate the overlap between adherence to physical activity, sleep, and sedentary behavior recommendations.

All analyses were conducted using R software (version 4.3.2) and RStudio (version 2023.12.1+402), with statistical significance set at two-sided *p* ≤ 0.05.

## 3. Results

Table 1 presents the descriptive characteristics of the participants (N = 671). Most participants were aged ≤18 years (n = 472; 70.3%). The distribution by sex was nearly equal, with 336 females (50.1%) and 335 males (49.9%). Most participants identified as heterosexual (n = 614; 91.5%), and the majority were Caucasian (n = 629; 93.7%). A large proportion were single (n = 483; 72.0%). Student accommodation was reported by 140 participants (20.9%), and current employment was reported by 93 participants (13.9%). Regarding the academic program, 266 participants (39.6%) were enrolled in health sciences. Overweight or obesity (BMI ≥ 25 kg/m^2^) was present in 106 participants (15.8% of 669 with valid BMI; median BMI 21.9 kg/m^2^). Mental health problems were reported by 67 participants (10.0%), and physical health problems were reported by 181 participants (27.0%).

**Figure 1:**
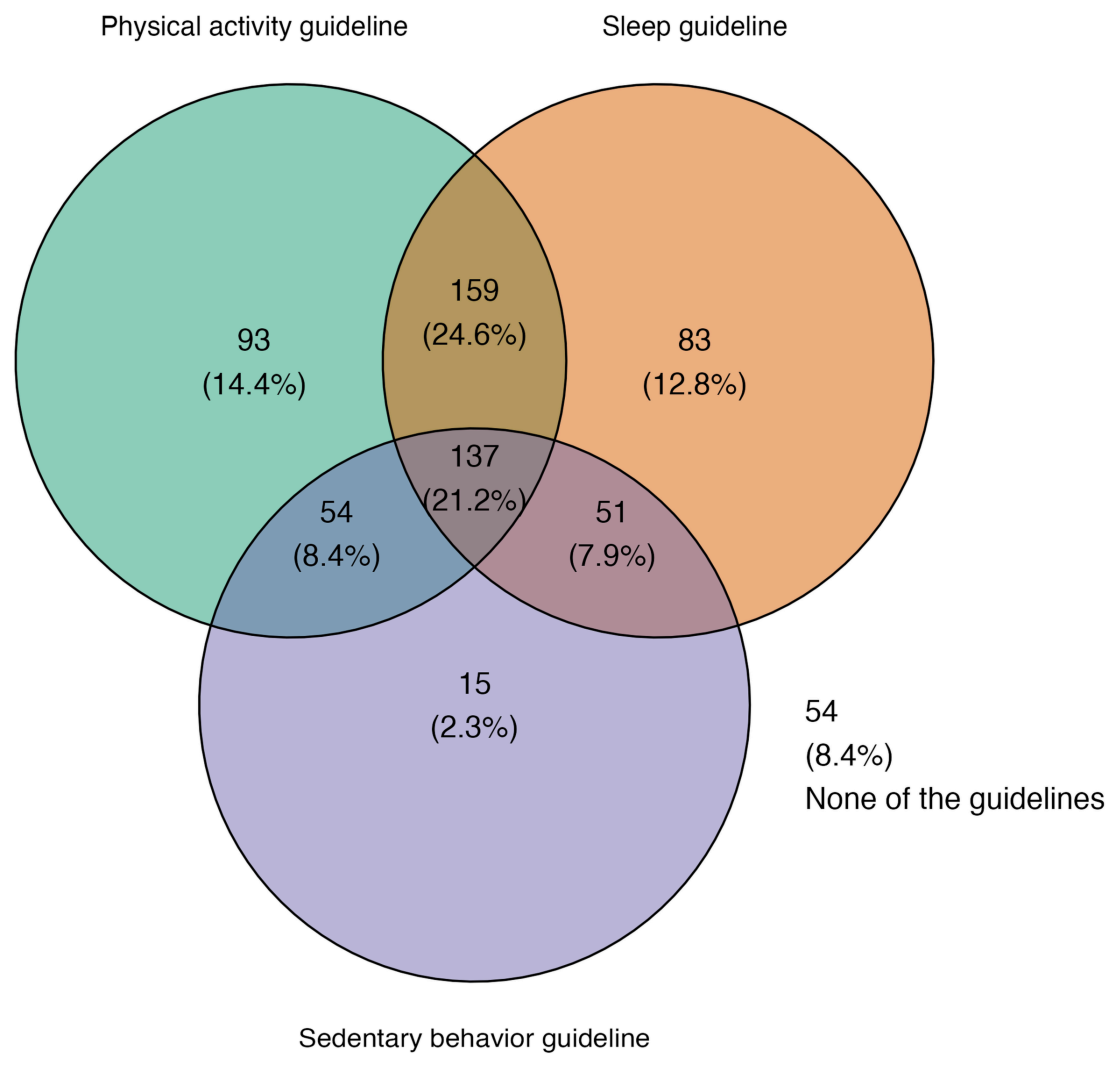
Venn diagram of adherence to the physical activity, sleep, and sedentary behavior components of the 24 h movement guidelines among first-year university students with complete guideline data (n = 646). Sedentary behavior adherence required both ≤8 h/day total sedentary time and ≤3 h/day recreational screen time.

Table 2 shows the descriptive data of the 24 h movement behaviors among the university students examined. The median time spent in moderate-to-vigorous physical activity (MVPA) was 240.0 min per week (IQR 120.0 to 660.0). Median recreational screen time was 180.0 min per day (IQR 120.0 to 300.0), and median sleep duration was 420.0 min per night (IQR 390.0 to 480.0). Among participants with complete data on all three components (n = 646), individual adherence was 68.6% for MVPA, 66.6% for sleep, and 39.8% for the combined sedentary recommendation (total sedentary ≤ 8 h/day and recreational screen ≤ 3 h/day; screen-only ≤ 3 h/day = 52.3%). When analyzed jointly, 137 students (21.2%) met all three 24 h movement guidelines. A total of 40.9% met two guidelines, 29.6% met one, and 8.4% did not meet any of the recommendations (Figure 1).

Table 3 presents the proportion of participants meeting combinations of the 24 h movement guidelines, stratified by sociodemographic characteristics. Meeting all three guidelines was more frequent among participants aged 18 years (23.1%) than among those aged >18 years (16.7%). Males showed higher adherence (25.2%) than females (17.4%). Students who identified as heterosexual showed higher adherence (22.2%) than LGBTQ+ students (10.5%). Adherence was similar between non-single (21.4%) and single students (21.1%). Finally, Caucasian participants showed higher adherence (21.5%) than non-Caucasian peers (17.1%).

Table 4 presents the proportion of participants meeting combinations of the 24 h movement guidelines, stratified by academic and labor characteristics. Adherence was higher among students enrolled in health sciences (24.1%) than among those in non-health sciences (19.3%). Adherence was similar between participants not living in student accommodation (21.4%) and those living in student accommodation (20.6%). Likewise, adherence was similar between students who were not working (21.4%) and those who were working (20.0%).

Table 5 presents the proportion of participants meeting combinations of the 24 h movement guidelines, stratified by physical and psychological characteristics. Overweight/obesity (BMI ≥ 25 kg/m^2^) was present in 15.8% of participants with valid anthropometric data. Participants with no mental health problems showed higher adherence than those with a lifetime mental health diagnosis (only 4 of 64 diagnosed students with complete guideline data met all three). Students with no physical health problems showed slightly higher adherence than those with physical health problems. Stratified percentages are descriptive; inferential interpretation relies on the multivariable model (Table 6). According to the multivariable logistic regression (Table 6; n = 644; 136 events), male students had higher odds of meeting all three 24 h movement guidelines compared with female students (aOR = 1.65; 95% CI 1.10 to 2.47; *p* = 0.016). Associations for age > 18 years (aOR = 0.64; 95% CI 0.39 to 1.03; *p* = 0.069) and non-health sciences enrollment (aOR = 0.69; 95% CI 0.46 to 1.03; *p* = 0.069) were borderline and not statistically significant at α = 0.05. Students with a lifetime mental health diagnosis had lower odds of adherence (aOR = 0.28; 95% CI 0.08 to 0.72; *p* = 0.018); Firth-penalized sensitivity analysis yielded aOR = 0.32 (95% CI 0.10 to 0.77). Overweight/obesity was not associated with adherence (aOR = 0.98; 95% CI 0.55 to 1.66; *p* = 0.929). When age was entered continuously, the age association was not significant (aOR = 0.94 per year; 95% CI 0.83 to 1.05; *p* = 0.335).

## 4. Discussion

Our findings suggest that approximately one in five students with complete guideline data (21.2%; 137/646) met all three 24 h movement guidelines, indicating relatively low adherence in this first-year university sample. This proportion is broadly consistent with previous studies conducted in similar settings. For instance, Bu et al. [20] reported a 27.0% adherence rate among 1846 Chinese university students, while Pengpid and Peltzer [18] found that only 11.7% of students across five countries met all three recommendations. These figures suggest that integrated movement behaviors remain suboptimal among many young adults. Conversely, our results contrast with those of Contini et al. [24], who found that just 0.2% of a sample of Canadian undergraduate students met the 24 h movement guidelines; their study applied a more stringent set of criteria, incorporating additional components such as muscle-strengthening activities and specific sleep and screen-time subdomains, which may partially explain the discrepancy. Collectively, these findings suggest that, regardless of country or methodological differences, a substantial proportion of university students do not meet integrated recommendations for physical activity, sedentary behavior, and sleep [13,14,15,16,25,26].

Additionally, our findings indicate that male students are more likely to meet the 24 h movement guidelines, which aligns with previous research conducted among both adolescent and university populations. Previous literature has consistently shown that males tend to report greater adherence to physical activity, sleep and screen time recommendations [13,18,20]. Although studies in younger populations have not always found sex-based differences in overall adherence [27], such disparities appear to become more pronounced during young adulthood, particularly in relation to physical activity levels [28]. This association may partly reflect sociocultural factors related to sex: males are often encouraged to engage in vigorous physical activity from early ages, while females may face stronger social norms and greater barriers, such as lower perceived safety, reduced self-efficacy, or time constraints [29]. Sedentary behavior patterns also tend to differ by sex, which may affect adherence to the integrated movement guidelines.

In the primary median-split model, students aged >18 years had borderline lower odds of meeting all three guidelines, but this association was not significant when age was modeled continuously (aOR 0.94 per year; 95% CI 0.83 to 1.05). Because all participants were first-year students and most were 18–19 years old, age-related differences should be interpreted cautiously and may reflect residual confounding (e.g., employment) rather than “mature-age” university pathways. We therefore avoid speculative explanations invoking caregiving burdens or social disengagement as primary mechanisms in this sample.

Students enrolled in health sciences programs showed a borderline association with higher adherence compared with peers from other academic disciplines in the multivariable model (non-health sciences aOR = 0.69; 95% CI 0.46 to 1.03; *p* = 0.069), which did not reach statistical significance at α = 0.05. The literature on this association remains inconclusive and, to some extent, contradictory [30,31,32]. For instance, studies in Spain and Croatia have shown that health sciences students report higher levels of physical activity, better dietary habits, and less screen time compared to those from other fields [30,31]. In contrast, a large study across 17 low- and middle-income countries found no significant differences in health risk behaviors between health and non-health science students [32]. These discrepancies may reflect contextual variations across countries and samples. Health sciences programs often include practical components (e.g., physical activity or nutrition workshops) and may promote higher health literacy [33], which could encourage healthier behaviors compared with students in non-health disciplines; however, our data support only a tentative, non-significant association and should not be over-interpreted.

Our findings also showed that students with a diagnosed mental health problem were less likely to adhere to all three 24 h movement guidelines. Although previous studies have reported associations between greater adherence and better mental health outcomes [13,25,26,27], the cross-sectional nature of our data does not allow us to determine whether mental health problems precede lower adherence, follow from it, or reflect shared underlying factors. We therefore frame this finding as a cross-sectional co-occurrence. Event counts among diagnosed students who met all three guidelines were very small (n = 4), so estimates remain uncertain despite Firth penalization. Symptoms such as low motivation, fatigue, sleep disturbances, and perceived barriers to physical activity [34,35,36,37,38] may coexist with lower adherence, but longitudinal studies are needed to clarify these relationships.

This study has several limitations that must be acknowledged. First, its cross-sectional design prevents the establishment of causal relationships between adherence to movement behaviors and potential associated factors (correlates). Second, all data were self-reported, which may introduce recall bias and social desirability bias, particularly in behaviors such as total sedentary time, recreational screen use, physical activity, or sleep duration; behaviors were assessed with PAS-2.1S rather than device-based measures. Furthermore, the lack of objective measurements, such as accelerometry for physical activity and sleep, may limit the accuracy of the estimates. Third, the sample comprises first-year students from one Spanish university recruited by convenience sampling and is not nationally representative. Fourth, the binary lifetime mental health diagnosis variable cannot capture symptom severity, diagnostic subtype, or subthreshold distress and may have limited sensitivity/specificity relative to validated symptom scales; sparse events among diagnosed students who met all three guidelines (n = 4) increase uncertainty. Despite these limitations, the findings have practical implications for hypothesis generation: integrated 24 h movement behavior interventions may warrant evaluation in university settings, with attention to subgroups that showed lower adherence in this sample (e.g., female students and individuals with mental health problems), without implying confirmed high-risk targeting from causal evidence.

## 5. Conclusions

In this cross-sectional study of first-year students from a single Spanish university, adherence to all three 24 h movement guidelines was relatively low (21.2%; 137/646 with complete guideline data). Male sex was associated with higher odds of adherence, and a lifetime mental health diagnosis was associated with lower odds; age and academic discipline showed only borderline associations. Because of the study design and sampling limitations, these associations should be interpreted as correlates, not causal determinants. Longitudinal research in more representative samples is needed to evaluate whether integrated approaches are feasible and whether mental health status and guideline adherence are related over time.

## Figures and Tables

**Table 1:** Descriptive data of the covariates of the study participants.

Variable	N = 671
Age group	
18	472 (70.3%)
>18	199 (29.7%)
Sex	
Female	336 (50.1%)
Male	335 (49.9%)
Sexual orientation	
LGBTQ+	57 (8.5%)
Heterosexual	614 (91.5%)
Race/ethnicity	
Caucasian	629 (93.7%)
Non-Caucasian	42 (6.3%)
Marital status	
Non-single	188 (28.0%)
Single	483 (72.0%)
Student accommodation	140 (20.9%)
Work status	93 (13.9%)
Degree program	
Health sciences	266 (39.6%)
Non-health sciences	405 (60.4%)
Overweight/obesity status	
No overweight/obesity	563 (84.2%)
Overweight/obesity	106 (15.8%)
Missing	2
Mental health	
No mental health problem	604 (90.0%)
Mental health problem	67 (10.0%)
Physical health	
No physical health problem	490 (73.0%)
Physical health problem	181 (27.0%)

Data was expressed as number (percentage). LGBTQ+, lesbian, gay, bisexual, transgender, queer/questioning, and other sexual orientations including pansexual and other non-heterosexual identities.

**Table 2:** Descriptive data of the 24 h movement behaviors of the study participants.

Variable	N = 671
LPA (min)	150.0 (90.0, 300.0)
Missing	19
MPA (min)	90.0 (30.0, 240.0)
Missing	15
VPA (min)	120.0 (0.0, 360.0)
Missing	16
MVPA (min)	240.0 (120.0, 660.0)
Missing	18
PA (min)	480.0 (240.0, 903.8)
Missing	23
PA guideline	
Meeting	447 (68.5%)
Non-meeting	206 (31.5%)
Missing	18
Sitting time (min)	420.0 (300.0, 480.0)
Missing	11
Screen time (min)	180.0 (120.0, 300.0)
Missing	18
SB guideline	
Meeting	262 (40.1%)
Non-meeting	391 (59.9%)
Missing	18
SD (min)	420.0 (390.0, 480.0)
Missing	9
SD guideline	
Meeting	441 (66.6%)
Non-meeting	221 (33.4%)
Missing	9
Twenty-four-hour guidelines	
None of the guidelines	54 (8.4%)
One guideline	191 (29.6%)
Two guidelines	264 (40.9%)
All three guidelines	137 (21.2%)
Missing	25

Median (interquartile range) or number (percentage). LPA, light physical activity; MPA, moderate physical activity; MVPA, moderate-to-vigorous physical activity; PA, physical activity; SB, sedentary behavior (meeting requires total sedentary time ≤ 8 h/day and recreational screen time ≤ 3 h/day); SD, sleep duration; VPA, vigorous physical activity. Combined 24 h guideline categories use complete cases (n = 646); 25 participants were missing one or more guideline components. Per-variable percentages use available cases for that variable; percentages reported in the text for participants with complete guideline data (n = 646) therefore differ slightly.

**Table 3:** Proportion of participants meeting combinations of the 24 h movement guidelines for physical activity, sedentary behavior and sleep, stratified by age group, sex, sexual orientation, marital status, and race/ethnicity.

Variable	Category	PA Only	Sleep Only	SB Only	PA + Sleep	PA + SB	Sleep + SB	All Three	None
Age group	18 years	60 (13.2%)	56 (12.3%)	11 (2.4%)	119 (26.2%)	33 (7.3%)	32 (7.0%)	105 (23.1%)	38 (8.4%)
	>18 years	33 (17.2%)	27 (14.1%)	4 (2.1%)	40 (20.8%)	21 (10.9%)	19 (9.9%)	32 (16.7%)	16 (8.3%)
Sex	Male	56 (17.6%)	27 (8.5%)	5 (1.6%)	94 (29.6%)	32 (10.1%)	14 (4.4%)	80 (25.2%)	10 (3.1%)
	Female	37 (11.3%)	56 (17.1%)	10 (3.0%)	65 (19.8%)	22 (6.7%)	37 (11.3%)	57 (17.4%)	44 (13.4%)
Sexual orientation	Heterosexual	85 (14.4%)	70 (11.9%)	15 (2.5%)	151 (25.6%)	49 (8.3%)	47 (8.0%)	131 (22.2%)	41 (7.0%)
	LGBTQ+	8 (14.0%)	13 (22.8%)	0 (0.0%)	8 (14.0%)	5 (8.8%)	4 (7.0%)	6 (10.5%)	13 (22.8%)
Marital status	Single	71 (15.3%)	54 (11.6%)	10 (2.2%)	114 (24.6%)	39 (8.4%)	38 (8.2%)	98 (21.1%)	40 (8.6%)
	Non-single	22 (12.1%)	29 (15.9%)	5 (2.7%)	45 (24.7%)	15 (8.2%)	13 (7.1%)	39 (21.4%)	14 (7.7%)
Race/ethnicity	Caucasian	86 (14.2%)	77 (12.7%)	12 (2.0%)	153 (25.3%)	52 (8.6%)	48 (7.9%)	130 (21.5%)	47 (7.8%)
	Non-Caucasian	7 (17.1%)	6 (14.6%)	3 (7.3%)	6 (14.6%)	2 (4.9%)	3 (7.3%)	7 (17.1%)	7 (17.1%)

PA, physical activity; SB, sedentary behavior. Values are n (%).

**Table 4:** Proportion of participants meeting combinations of the 24 h movement guidelines for physical activity, sedentary behavior and sleep, stratified by degree program, student accommodation status, and working status.

Variable	Category	PA Only	Sleep Only	SB Only	PA + Sleep	PA + SB	Sleep + SB	All Three	None
Degree program	Health sciences	39 (15.2%)	31 (12.1%)	5 (1.9%)	58 (22.6%)	18 (7.0%)	19 (7.4%)	62 (24.1%)	25 (9.7%)
	Non-health sciences	54 (13.9%)	52 (13.4%)	10 (2.6%)	101 (26.0%)	36 (9.3%)	32 (8.2%)	75 (19.3%)	29 (7.5%)
Accommodation	Student accommodation	18 (13.7%)	18 (13.7%)	8 (6.1%)	23 (17.6%)	10 (7.6%)	7 (5.3%)	27 (20.6%)	20 (15.3%)
	No student accommodation	75 (14.6%)	65 (12.6%)	7 (1.4%)	136 (26.4%)	44 (8.5%)	44 (8.5%)	110 (21.4%)	34 (6.6%)
Working status	Working	16 (17.8%)	9 (10.0%)	1 (1.1%)	19 (21.1%)	17 (18.9%)	6 (6.7%)	18 (20.0%)	4 (4.4%)
	Not working	77 (13.8%)	74 (13.3%)	14 (2.5%)	140 (25.2%)	37 (6.7%)	45 (8.1%)	119 (21.4%)	50 (9.0%)

PA, physical activity; SB, sedentary behavior. Values are n (%).

**Table 5:** Proportion of participants meeting combinations of the 24 h movement guidelines for physical activity, sedentary behavior and sleep, stratified by overweight/obesity, mental health status, and physical health status.

Variable	Category	PA Only	Sleep Only	SB Only	PA + Sleep	PA + SB	Sleep + SB	All Three	None
Overweight/obesity	Yes	23 (22.1%)	9 (8.7%)	3 (2.9%)	21 (20.2%)	16 (15.4%)	6 (5.8%)	21 (20.2%)	5 (4.8%)
	No	70 (13.0%)	74 (13.7%)	12 (2.2%)	138 (25.6%)	38 (7.0%)	44 (8.1%)	115 (21.3%)	49 (9.1%)
Mental health	Mental health problem	10 (15.6%)	14 (21.9%)	2 (3.1%)	12 (18.8%)	12 (18.8%)	2 (3.1%)	4 (6.2%)	8 (12.5%)
	No mental health problem	83 (14.3%)	69 (11.9%)	13 (2.2%)	147 (25.3%)	42 (7.2%)	49 (8.4%)	133 (22.9%)	46 (7.9%)
Physical health	Physical health problem	33 (19.2%)	24 (14.0%)	2 (1.2%)	42 (24.4%)	20 (11.6%)	9 (5.2%)	31 (18.0%)	11 (6.4%)
	No physical health problem	60 (12.7%)	59 (12.4%)	13 (2.7%)	117 (24.7%)	34 (7.2%)	42 (8.9%)	106 (22.4%)	43 (9.1%)

PA, physical activity; SB, sedentary behavior. Values are n (%).

**Table 6:** Multivariable logistic regression examining associated factors of adherence to the 24 h movement guidelines among first-year university students (Universidad Loyola Andalucía; complete-case n = 644).

Predictor	aOR	95% CI	*p* Value
Age group			
≤18 years old	Ref.		
>18 years old	0.64	0.39 to 1.03	0.069
Sex			
Female	Ref.		
Male	1.65	1.10 to 2.47	0.016
Sexual orientation			
LGBTQ+	Ref.		
Heterosexual	1.81	0.80 to 4.88	0.191
Race/ethnicity			
Caucasian	Ref.		
Non-Caucasian	0.80	0.31 to 1.81	0.618
Marital status			
Non-single	Ref.		
Single	0.91	0.59 to 1.41	0.655
Living in a student accommodation			
Yes	Ref.		
No	1.08	0.67 to 1.79	0.750
Work status			
Yes	Ref.		
No	0.99	0.55 to 1.85	0.982
Degree program			
Health sciences	Ref.		
Non-health sciences	0.69	0.46 to 1.03	0.069
Overweight/obesity status			
No overweight/obesity	Ref.		
Overweight/obesity	0.98	0.55 to 1.66	0.929
Mental health			
No mental health problem	Ref.		
Mental health problem	0.28	0.08 to 0.72	0.018
Physical health			
No physical health problem	Ref.		
Physical health problem	0.80	0.50 to 1.25	0.340

CI, confidence interval; LGBTQ+, lesbian, gay, bisexual, transgender, queer/questioning, and other sexual orientations including pansexual and other non-heterosexual identities; Ref., reference.

## Data Availability

The data supporting the findings of this study are not publicly available due to ethical and data protection restrictions. Data may be made available by the corresponding author upon reasonable request, subject to appropriate ethical and institutional approval and applicable data protection requirements.

## Abbreviations

The following abbreviations are used in this manuscript:

MVPA	Moderate- to vigorous-intensity physical activity
BMI	Body mass index
UNILIFE-M	UNIversity Students’ LIFEstyle Behaviours and Mental Health
PAS-2.1S	Physical Activity Scale 2.1 Spanish version
LPA	Light physical activity
MPA	Moderate physical activity
VPA	Vigorous physical activity
WHO	World Health Organization
ADHD	Attention-deficit/hyperactivity disorder
ASD	Autism spectrum disorder
Q–Q	Quantile–quantile plots
IQR	Interquartile range
aOR	Adjusted odds ratio
CI	Confidence interval
